# Chemical Characterisation and in Vitro Gas Production Kinetics of Eight Faba Bean Varieties

**DOI:** 10.3390/ani10030398

**Published:** 2020-02-28

**Authors:** Alessandra Pelagalli, Nadia Musco, Nikita Trotta, Monica I. Cutrignelli, Antonio Di Francia, Federico Infascelli, Raffaella Tudisco, Pietro Lombardi, Alessandro Vastolo, Serena Calabrò

**Affiliations:** 1 Department of Advanced Biomedical Sciences, University of Napoli Federico II, 80100 Napoli, Italy; alessandra.pelagalli@unina.it; 2 Department of Veterinary Medicine and Animal Production, University of Napoli Federico II, 80100 Napoli, Italy; nadia.musco@unina.it (N.M.); federico.infascelli@unina.it (F.I.); raffaella.tudisco@unina.it (R.T.); pietro.lombardi@unina.it (P.L.); alessandro.vastolo@unina.it (A.V.); serena.calabro@unina.it (S.C.); 3 Centro di ricerca Politiche e Bio-economia R, CREA, Consiglio per la ricerca in agricoltura e l’analisi economica agraria (CREA), 80143 Napoli, Italy; nikita.trotta@crea.gov.it; 4 Department of Agricultural Sciences, University of Napoli Federico II, 80100 Napoli, Italy; antonio.difrancia@unina.it

**Keywords:** protein source, ruminant, in vitro fermentation, degradability, volatile fatty acids

## Abstract

**Simple Summary:**

The demand of vegetable protein is currently very high, both for human and animal nutrition. Soybean meal is the most used protein source in ruminant nutrition. Many Leguminosae seeds (i.e., faba bean, lupin, proteic pea), rich in protein and energy, are considered a valid alternative, especially in the organic production system. In this paper the physical and nutritional characteristics of eight varieties of *Vicia Faba* bean (four local and four commercial) were evaluated. To evaluate the digestive utilization an in vitro trial was carried out, incubating each substrate with an inoculum made up of bovine buffered rumen liquor for 48 h at 39 °C under anaerobiosis. The gas produced within the incubation period was registered, the dry matter digestibility and volatile fatty acid at the end of fermentation were determined. The results of this investigation confirm the possibility of using local faba bean varieties in ruminant nutrition with the advantages that, being local natural resources, they are better adapted to the climate and agronomic conditions and limit the environmental impact.

**Abstract:**

Faba bean is an important vegetable protein source for ruminant diets. This research aimed to compare the nutritional characteristics of four commercial and four local cultivars in order to better characterise the local ones and promote their use in animal nutrition. The seeds’ weight and the chemical composition, including starch and the energy, was evaluated. The in vitro fermentation characteristics were studied for 48 h using bull’s rumen fluid as inoculum. All the varieties showed the values’ weight corresponding to the specific botanical typology. The varieties significantly differed for protein, starch and lignin (*p* < 0.01) and structural carbohydrates (*p* < 0.05) concentration. No significant differences were observed for energy content. All the in vitro fermentation parameters resulted significantly different among the varieties. Organic matter degradability ranged between 89.9% and 85.1% and the potential gas production from 367 to 325 mL/g. The Pearson’s analysis showed significant correlation between morphological characteristics, chemical data and in vitro fermentation parameters. In conclusion, this investigation confirms the possibility of using local faba bean varieties (i.e., Aquino, Castrocielo, 13#5, 4#4) in ruminant nutrition with the advantage that, being local natural resources, they are better adapted to the climate and agronomic conditions and limit environmental impact.

## 1. Introduction

Grain legumes, both rich in protein and energy, have numerous and valuable uses in feed materials, as well as in food production [1]. In animal nutrition, many Leguminosae seeds (i.e., faba bean, lupin, proteic pea) are mainly considered a valid protein source, but the high starch content should also be taken into consideration [2]. These seeds are used as an alternative to soybean meal in the organic production system [3] and their cultivation is promoted by the European Union [4].

Faba bean (*Vicia faba* spp.) is grown worldwide as a source of protein and starch for humans and animals [5]. Its production is widespread in China, followed by Australia, France and Egypt [6,7]. The European contribution to global production is only 14.4 %, but the average yield registered in the Mediterranean countries is nearly double that of anywhere else. *Vicia faba* L. is an auto-diploid plant and it is classified into three main botanical types according to seed dimensions (g/n of seeds): major Herz, higher than 1000/1000, minor Beck, up to 700/1000 and equina Pers. 700–1000/1000 [8]. The first two types are gene pools of the Central and Northwest European varieties, while equina is more present in Mediterranean European varieties. Many other systematic classifications (e.g., based on the taxonomic levels) are known, but none of them has been completely defined. Moreover, it is important to consider how the environmental conditions as well as the origin can modify seeds by altering their dimensions and reducing the differences among varieties [6]. Like others legume seeds, faba bean improves soil fertility and reduces the nitrogen fertilization requirements by fixing atmospheric nitrogen in symbiosis with the soil rhizobia bacteria [9], thus playing an important role in crop rotation. The faba bean has been successfully used in Mediterranean areas as a high-protein concentrate for ruminants. Brunschwig and Lamy [10] showed that the use of 30% of ground faba beans in the concentrate for dairy cows did not alter feed consumption and milk production, in terms of yield, and milk quality (i.e., protein, fat, etc.). It is suitable also for growing animals: Leitgeb and Lettner [11] reported that the use of faba beans did not decrease feed consumption and did not alter animal growth and the carcass composition. In previous investigations, the faba bean is shown contributing to improving the local animal products (i.e., lower cholesterol and saturated fatty acid contents) such as meat from buffalo [12] and from Marchigiana young bulls [13,14].

In the last few years, according to EU strategy [15], many projects have been promoted in Italy in order to reduce the continuous loss of agronomical biodiversity. Moreover, the local production of some Leguminosae seeds should be valorised. The aim of the present research was to compare the morphological and nutritional characteristics of eight faba bean varieties, including four local varieties grown in the Mediterranean area, and four commercial varieties recorded in the National Agricultural Varieties Register. All the varieties were evaluated for morphological aspects, chemical composition and in vitro fermentation patterns. The hypothesis was that the local faba bean varieties could present nutritional characteristics comparable to the commercial ones and, consequently, be useful for ruminant nutrition.

## 2. Materials and Methods

### 2.1. Experimental Design

The eight varieties of faba bean investigated in the present study were selected in different Italian Regions among the three main types (equina, minor and major) by the researchers of the Centro di Ricerca Orticoltura e Florovivaismo (CREA-ORT, Pontecagnano, SA, Italy): four local varieties (Aquino, Castrocielo, 13#5, 4#4) and four commercial ones (Bolivia, Chiaro di Torre Lama, Sikelia and Aguadulce Supersimonia), as reported in Table 1. The commercial varieties were chosen as control for their similarity in morphological aspects with the local varieties. At CREA-ORT (40°38’36"60 N, 14°52’27"48 E, 34 m a.s.l.) in a 192 m^2^ (32.0 m × 6.0 m), a field experiment was conducted to produce grains to be evaluated. For each variety, four randomized plots (5 m^2^) were sown with 30 seeds positioned at 6–8 cm of depth in rows of 45 cm. During plant cultivation no fertilizing or pesticide treatments were carried out, except during the first growth stage, when an insecticide treatment, based on pirimicarb, was used. The seeds produced by the field trial were harvested from each plot and weighed in order to evaluate the seed yield and size. Subsequently, in the laboratory mixing two-by-two the seeds for each variety (two plots/variety), two aliquots were obtained. Then, the obtained samples were dried and characterized for chemical composition and the in vitro fermentation characteristics.

### 2.2. Seed Size

The morphological analysis of the faba seeds was performed according to the International Union for the protection of New Plant Varieties [16], evaluating the weight of 1000 seeds (g).

### 2.3. Nutritional Evaluation of Seeds

At the Department of Veterinary Medicine and Animal Production (Napoli, Italy) the seeds were milled at 1.1 mm screen (SM 100, Retsch, Haan, Germany) to determine dry matter (DM), crude protein (CP) and ash contents according to official procedures [17]: ID members 934.01, 954.01 and 942.05, respectively. Moreover, the neutral detergent fibre (aNDFom, adding sodium sulphite and heat resistant alpha-amylase, Ankom Macedon NY), the acid detergent fibre (ADF) and the lignin (ADL) were determined [18]. The starch content was determined through polarimetric detection (Polax L, Atago, Tokyo, Japan) according to the official procedure [19]. The energy content as metabolizable energy (ME, MJ/kg) was estimated according to INRA system as following [20]:

(43.3 x CP x degCP) + (77.2 x EE x degEE) + (35.9 x CF x degCF) + (36.3 x NFE x degNFE)(1)

where: EE, CF and NFE are crude protein, ether extract, crude fiber and nitrogen free extract content (%), respectively; deg represent the degradability coefficients of each parameter proposed by the INRA system.

### 2.4. In Vitro Fermentation Characteristics

The fermentation characteristics and kinetics were studied using the in vitro gas production technique as proposed by Theodorou [21]. Two gas runs were conducted under similar experimental conditions within two weeks. All the samples (varieties) were incubated as substrates at 39 °C under anaerobic conditions with buffered rumen fluid [22]. Faba bean samples were weighed (1.000 g ± 0.002) in triplicate in 120 mL serum flasks with anaerobic medium (74 mL). Three flasks without substrate were incubated to correct organic matter degradability and gas volume. The rumen fluid was collected in a pre-warmed thermos from six/run bovine fasted young bulls (*Bos taurus*), regularly slaughtered in an authorized facility in accordance with current animal welfare legislation [23]. For 15 days before slaughter young bulls were fed 13 kg of dry matter/die of standard diet (NDF 45.5 and CP 12.0% DM) containing on dry matter basis 20% of corn silage, 20% of mixed hay and 40% of commercial concentrate (i.e., beet pulp, wheat middling, wheat flour middling, maize gluten, soya bean meal) [24]. All procedures involving animals were approved by the Ethical Animal Care and Use Committee of the University of Napoli Federico II (Prot. 2019/0013729 of 08/02/2019). The collected material was rapidly transported to a pre-warmed thermos in the laboratory, where it was pooled, flushed with CO_2_, filtered through cheesecloth and added to each flask (5 mL) within 1 h of collection. All the steps were carried out at 39 °C and under insufflations of CO_2_ to maintain anaerobic conditions. Gas production was recorded 17 times, during the 48 h of incubation, using a manual pressure transducer (Cole and Parmer Instrument Co., Vernon Hills, IL, USA). At this time the fermentation was stopped by cooling (4 °C) and the degraded organic matter (dOM, %) was determined by the differences between the incubated OM and the residual OM, obtained filtering the fermentation liquor throughout pre-weighed sintered glass crucibles (Schott Duran, Mainz, Germany, porosity #2) and successively burnt at 550 °C. An aliquot of fermentation liquor was taken to measure the pH using a pH-meter (ThermoOrion 720 A+, Fort Collins, CO, USA). Volatile fatty acids (VFA, mM/g) were determined through gas chromatography (GC Focus AI 3000, Thermo Scientific, Waltham, MA, USA) equipped with a fused silica capillary column SACTM-5, Supelco, 30 m x 0.25 mm x 0.25 μm film thickness using external standard solution (acetate, propionate, butyrate, iso-butyrate, valerate and iso-valerate) [24].

### 2.5. Statistical Analysis

The cumulative volume of gas produced at 48 h was related to incubate OM (OMCV, mL/g). For each flask the gas production profiles were processed with the following model [25]:

(2) $$G=\frac{A}{\left[1+\left(\frac{C^{B}}{t^{B}}\right)\right]}$$

where, G is the total gas produced (mL/g of incubated OM) at time t (h), A the asymptotic gas production (mL/g of incubated OM), B (h) the time at which one-half of the asymptote is reached, and C the curve switch. Maximum fermentation rate (R_max_, mL/h) and time at which it occurs (T_max_, h) were also calculated:

(3) $$Rmax=\frac{A*B^{C}*B*Tmax^{\left(B-1\right)}}{\left[1+{\left(C^{B}*Tmax^{-B}\right)}^{2}\right]}$$

(4) $$Tmax=C*{\left[\frac{\left(B-1\right)}{\left(B+1\right)}\right]}^{1/B},$$

Seed size, chemical data, fermentation characteristics, and model parameters were statistically compared between faba bean varieties [JMP® 1989–2007, Version 9.0, Cary (NC): SAS Institute Inc.] using the model:

(5) $$y_{ij}=\mu +Var_{i}+\varepsilon_{ij},$$

where y is the single datum, μ the mean, Var the variety (substrate) effect (i = 8) and ε the error term. The *t* test was used to verify the differences between means, considering for each variety four subsamples (e.g., plots) for seed size; two subsamples (mixing two-per-two plots) for chemical composition and four subsamples (the same samples of chemical composition used for two gas runs). The correlations among seed characteristics, chemical composition and in vitro data (*p <* 0.05) were also studied using the same software.

## 3. Results

On average, the seeds’ yield in field for all the cultivated faba bean varieties was 395 ± 58 kg/ha (at 10% moisture). No significant differences (*p* > 0.05) were found between varieties.

### 3.1. Seeds Size

**Figure 1:**
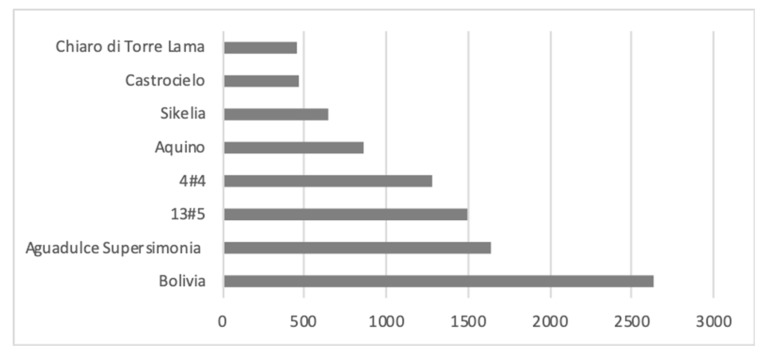
Weight of 1000 seeds of faba bean varieties (g).

Comparing the weights, which referred to 1000 seeds (Figure 1), it was possible to highlight that all the analysed varieties showed characteristics corresponding to the specific botanical typology: Chiaro di Torre Lama, Castrocielo and Sikelia weighed less than 700 g, corresponding to the minor type; the Aquino varieties showed a weight of 856 g, corresponding to the equina type while all the remaining varieties showed weights higher than 1000 g. The weight of the Bolivia variety (2633 g) was particularly high.

### 3.2. Chemical Composition

The varieties significantly (*p <* 0.01, *p <* 0.05) differed in their chemical composition (Table 2). In particular, on DM basis the protein content was similar among the varieties, even if Aguadulce Supersimonia and 4#4 showed the highest (*p <* 0.01) values (293 and 290 g/kg, respectively). With regard to the structural carbohydrates, NDF on DM basis ranged from 145 and 228 g/kg in Bolivia and Sikelia, respectively and showed few statistical differences, whereas the lignin was significantly (*p <* 0.01) higher in 4#4 (46.7 g/kg). Instead, the starch content varied at a wider range (*p <* 0.01): Sikelia showed the highest value (438 g/kg) and 4#4 the lowest one (221 g/kg). The ash content slightly varied among varieties ranging from 35.6 g/kg in 13#5 and 45.1 g/kg in Bolivia. No significant differences were observed for energy content which varied from 10.9 to 11.9 MJ/kg.

### 3.3. In Vitro Fermentation Characteristics and Volatile Fatty Acids Production

**Figure 2:**
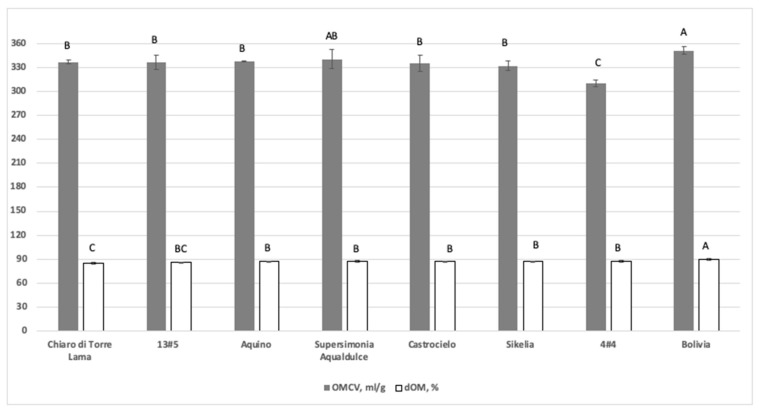
In vitro fermentation characteristics of faba bean varieties; dOM = organic matter degradability (% of incubated OM; *p* = 0.0007); OMCV = cumulative volume of gas related to incubated OM (mL/g; *p* = 0.0004). For each parameter: A–C = *p <* 0.01. Error bar = standard deviation.

All the fermentation parameters resulted as being significantly (*p <* 0.01) different among the varieties (Figure 2 and Table 3). All the varieties showed a percentage of degradability higher than 85% albeit by some significant differences (*p <* 0.01) among varieties were observed. The Bolivia variety showed the highest dOM value (89.9%) whereas the Chiaro di Torre Lama was the least degradable (dOM: 85.1%). The other varieties showed intermediate values. Regarding the gas production, 4#4 showed the lowest value (OMCV: 310 mL/g) whereas Bolivia and Sikelia the highest ones (351 and 332 mL/g, respectively). In most of the varieties, with the exception of the 4#4 and Sikelia a linear relation between dOM and OMCV was observed. The potential gas production varied little among the studied faba bean varieties, even if 4#4 presented the lowest value (325 mL/g; *p* < 0.01). The time at which the A/2 was reached (B parameter) varied from 13.7 h of the Aquino to 15.5 h of the 4#4 variety showing a mean value of 14.1 h ± 0.5. The maximum fermentation rate of Bolivia and Aquino reached the same maximum fermentation rate (R_max_: 18.9 ml/h) in a similar time (T_max_: 9.7 and 9.8 h, respectively). Sikelia showed a similar T_max_ value (9.4 h) with the lowest R_max_ (16.2 mL/h).

The pH values ranged from 6.89 to 6.94 in Castrocielo and Chiaro di Torre Lama, respectively, indicating that after 48 h the incubation was carried out adequately.

The volatile fatty acid (VFA) production (Table 4) was also significantly (*p <* 0.01) affected by the variety. With regard to the total VFA, Chiaro di Torre Lama showed the lowest value (75.8 mM/g; *p <* 0.01) mainly due to the lower production of acetate, isovalerate and butyrate, whereas Bolivia showed the highest VFA (98.0 mM/g) mainly due to the higher acetate, propionate and butyrate production in comparison to the other varieties. Overall, for the other varieties these parameters were similar.

The Pearson’s analysis (r) showed a significant correlation between the morphological characteristics, chemical data and in vitro fermentation parameters. Seed size affected positively the energy content (0.68; *p <* 0.05) and negatively NDF level (−0.89; *p <* 0.01). The structural carbohydrates negatively (*p <* 0.01) influenced the fermentation kinetics parameters (NDF vs. R_max_: −0.67 and ADF vs. T_max_: −0.80). Despite the low percentage of lignin in all the samples, this parameter negatively affected the OMCV (−0.88, *p <* 0.01). The starch fraction was positively correlated (*p <* 0.05) with A (0.63) and B (0.67).

## 4. Discussion

Like other legume seeds, *Vicia faba* spp. can be considered a valuable protein source for ruminants albeit the different varieties need to be better evaluated for their more appropriate use. For the varieties tested in our study, the seed size, chemical composition, as well as the in vitro fermentation characteristics, fall into the range reported in the literature [26,27]. Our data concerning protein content are in line with those reported by other authors [28,29], ranging from 24.2 to 37.2%. With the in sacco method, Faurie et al. [30] showed that more than 85% of the nitrogen is degraded in the rumen in 2 h resulting in a mean theoretical nitrogen degradability of 92% for three different faba bean cultivars. For this reason, in a previous study [13] we suggested associating a protein source richer in rumen un-degradable protein to faba beans immediately after weaning. The structural carbohydrates of faba beans had a lower NDF content (mean value: 19.6% DM) compared to a previous study [27] on six faba bean varieties (22.7% DM). In this study, the NDF content was quite variable (from 14.5% to 22.9% DM) in function of the varieties, similarly to the data obtained in 74 faba bean varieties (NDF from 13.4% to 26.4% DM) [29]. Except for the very high value recorded in variety 4#4, the ADL contents were quite low and similar to those registered in a previous study [27]. According to other authors [31,32], the starch content was quite high (from 22.1% to 43.8% DM), even if Duc et al. [29] found higher values (37.0% to 50.5% DM). The high energy content and the low structural carbohydrate level in the tested faba beans grains favored in vitro fermentation patterns in terms of degradability, kinetics, gas and VFA production. In a previous study [27], incubating in vitro six different varieties of faba beans (i.e., Irene, Lady, Scuro di Torre Lama, Chiaro di Torre Lama, ProtHABAT69 and Siconia) with rumen fluid from buffalo, higher degradability values (dOM mean values: 91.8% vs. 87.3%), but slower fermentation kinetics (T_max_ mean value: 13.2 vs. 9.5 h; R_max_ mean value: 8.7 vs. 17.7 mL/h) were registered. Azarfar et al. [33], incubating for 72 h processed grains of a variety of Vicia faba minor using in vitro gas production technique with rumen liquor from dairy cows, found a lower gas production value (OMCV mean value: 194 vs. 335 mL/g) and a lower volatile fatty acid production (acetate, propionate and butyrate: 68.0 vs. 79.7 mM/g). These data indicate that many factors could influence the in vitro fermentation pathway (i.e., varieties, donor animal species, incubation time). As reported in a previous study [2], the carbohydrates’ fractions differently affect the fermentation kinetics: starch promotes a more intense and rapid process, conversely structural carbohydrates cause a slower and less consistent fermentation. The particularly slow in vitro fermentation kinetics of the 4#4 variety (potential gas production lowest value, *p* < 0.001) was probably due to the high ADL content that has limited the access to the cell content by micro-organisms, reducing nutrients’ degradability and slowing down the fermentation rate; as evidenced, also by the negative correlation between lignin content and OMCV. No significant correlation was observed between crude protein and OMCV and dOM in contrast with a previous study [2] on lupine seeds. The different results could be probably due to more balanced ratio between protein and carbohydrates of faba bean compared to lupine.

The significant correlation between the seed size and some chemical parameters (NDF and energy) testify the lower incidence of structural carbohydrates in the mass units (1 kg) and the higher energy value of the varieties bigger in size. However, no correlation was observed between seed size and in vitro parameters. All these findings confirm the nutritional characteristics of *Vicia faba*, endorsing the results of several authors obtained in vivo on growing and dairy ruminants [10,11,12,13,14].

Comparing the local varieties with their morphologically homologous commercial ones (Aquino vs. Sikelia; Castrocielo vs. Chiaro di Torre Lama; 13#5 and 4#4 vs. Bolivia and Aguadulce Supersimonia), some interesting results emerged from the nutritional point of view. The enhancement of local varieties already evidenced by historical documentation [34] assumes greater importance. For example, the Aquino variety showed specific characteristics (i.e., high protein, starch and energy contents, low fiber amounts, high in vitro fermentability and VFA production).

## 5. Conclusions

The results of this investigation confirm the possibility of using local faba bean varieties in ruminant nutrition with the advantage that, being local natural resources, they are better adapted to the climate and agronomic conditions and limit the environmental impact. These varieties, such as the commercial ones, present different nutritional characteristics, that affect the in vitro fermentation kinetics, and could also influence their in vivo utilization.

In any event, their use as alternative protein sources to the most common extruded soybean meal needs further studies aimed at evaluating the possible presence of anti-nutritional factors (i.e., tannins, vicine, etc.) that could negatively affect their digestive utilization and/or positively influence the environmental sustainability reducing green-house gases (e.g., methane) production.

## Figures and Tables

**Table 1:** Harvesting area of the *Vicia faba* L. varieties used for the field experiment.

Variety	Type	Province and Region	Latitude North	Longitude East	Altitude [m a.s.l.]
Local					
Aquino	*Equina*	Frosinone, Lazio	41°29′	13°42′	110
Castrocielo	*Minor*	Frosinone, Lazio	41°31’	13°41’	226
4#4	*Major*	Salerno, Campania	40°40’	14°46’	54.3
13#5	*Major*	Salerno, Campania	40°40’	14°46	54.3
Commercial					
Bolivia	*Major*	Roma, Lazio	41°94’	12°67’	20
Chiaro di Torre Lama	*Minor*	Massa Carrara, Toscana	43°25’	13°25’	350
Sikelia	*Minor*	Catania, Sicilia	37°51’	15°07’	7
Aguadulce Supersimonia	*Major*	Arezzo, Toscana	43°49’	11.63’	296

**Table 2:** Chemical composition on dry matter basis of faba bean varieties.

Variety	CP	NDF	ADF	ADL	Starch	Ash	ME
	g/kg						MJ/kg
Local							
Aquino	273 ^B^	207 ^ab^	92.9 ^EF^	8.0 ^B^	390 ^B^	42.6 ^AB^	11.3
Castrocielo	264 ^BC^	222 ^a^	142 ^A^	6.7 ^B^	352 ^C^	37.5 ^EF^	11.5
13#5	250 ^D^	201 ^ab^	146 ^A^	10.1 ^B^	378 ^B^	35.6 ^F^	11.9
4#4	290 ^A^	201 ^ab^	120 ^B^	46.7 ^A^	221 ^E^	42.7 ^B^	11.2
Commercial							
Bolivia	254 ^CD^	145 ^c^	98.6 ^DE^	7.6 ^B^	298 ^D^	45.2 ^A^	11.8
Chiaro di Torre Lama	260 ^C^	199 ^ab^	88.1 ^E^	9.7 ^B^	375 ^BC^	38.1 ^DE^	11.2
Sikelia	245 ^D^	229 ^a^	104 ^CD^	8.7 ^B^	438 ^A^	41.6 ^BC^	10.9
Aguadulce Supersimonia	293 ^A^	168 ^bc^	111 ^BC^	9.1 ^B^	383 ^B^	40.0 ^CD^	11.9
RMSE	4.4	19.7	3.86	3.62	9.95	0.95	1.57
*p* value	<0.001	0.038	<0.001	<0.001	<0.001	<0.001	0.195

CP = crude protein; NDF = neutral detergent fiber; ADF = acid detergent fiber; ADL = acid detergent lignin; ME = metabolizable energy. Along the column: a–c = *p* < 0.05 and A–F = *p* < 0.01; RMSE = root mean square error.

**Table 3:** In vitro fermentation characteristics of faba bean varieties.

Variety	A	B	T_max_	R_max_
	mL/g	h	h	mL/h
Local				
Aquino	350 ^B^	13.7 ^DE^	9.8 ^ab^	18.9 ^A^
Castrocielo	355 ^AB^	14.4 ^B^	9.3 ^bc^	17.0 ^CDE^
13#5	353 ^B^	14.0 ^CD^	9.1 ^c^	17.3 ^BCD^
4#4	325 ^C^	13.5 ^E^	9.0 ^c^	16.8 ^DE^
Commercial				
Bolivia	367 ^A^	14.0 ^CD^	9.7 ^abc^	18.9 ^A^
Chiaro di Torre Lama	353 ^B^	14.2 ^BC^	10.1 ^a^	18.3 ^AB^
Sikelia	360 ^AB^	15.0 ^A^	9.4 ^bc^	16.2 ^E^
Aguadulce Supersimonia	360 ^AB^	14.0 ^CD^	9.2 ^bc^	17.9 ^ABC^
RMSE	8.4	0.3	0.4	0.8
*p* value	<0.001	<0.001	0.033	<0.001

A = potential gas production; B = time at which A/2 is formed; T_max_ = time at which maximum fermentation rate was reached; R_max_ = maximum fermentation rate. Along the column: a–c = *p* < 0.05 and A–E = *p <* 0.01. RMSE = root mean square error.

**Table 4:** pH values and concentration of volatile fatty acids after 48 h of in vitro incubation.

Variety	pH	Acetate	Propionate	Isobutyrate	Butyrate	Isovalerate	Valerate	VFA
		mM/g						
Local								
Aquino	6.92 ^B^	47.8 ^A^	15.8 ^A^	2.26 ^AB^	19.3 ^AB^	4.64 ^B^	2.51 ^AB^	92.3 ^AB^
Castrocielo	6.89 ^D^	46.3 ^AB^	13.7 ^BC^	2.00 ^BC^	20.1 ^AB^	4.30 ^BC^	2.00 ^AB^	88.4 ^AB^
13#5	6.92 ^BC^	47.5 ^AB^	15.0 ^AB^	2.05 ^BC^	17.9 ^B^	4.16 ^BCD^	2.40 ^AB^	89.1 ^AB^
4#4	6.90 ^D^	48.6 ^A^	14.8 ^AB^	1.95 ^C^	18.5 ^AB^	3.89 ^CD^	1.94 ^B^	89.7 ^AB^
Commercial								
Bolivia	6.91 ^CD^	51.0 ^A^	15.5 ^AB^	2.39 ^A^	22.0 ^A^	5.23 ^A^	1.95 ^AB^	98.0 ^A^
Chiaro di Torre Lama	6.94 ^A^	42.3 ^B^	12.0 ^C^	1.79 ^C^	13.5 ^C^	3.67 ^D^	2.55 ^A^	75.8 ^C^
Sikelia	6.92 ^BC^	47.8 ^A^	14.8 ^AB^	2.06 ^BC^	19.9 ^AB^	4.13 ^BCD^	2.38 ^AB^	91.0 ^AB^
Aguadulce Supersimonia	6.93 ^AB^	45.9 ^AB^	14.0 ^B^	1.98 ^C^	19.1 ^AB^	4.03 ^CD^	1.93 ^B^	87.0 ^B^
RMSE	0.90	4.49	1.59	0.23	3.14	0.47	0.52	9.35
*p* value	<0.001	0.105	0.005	0.003	0.036	<0.01	0.132	0.022

VFA = volatile fatty acids. Along the column: A–D = *p <* 0.01. RMSE = root mean square error.
